# Periostitis of the Pubic Symphysis in Inguinal and Femoral Hernia: An Uninterpreted Source of Chronic Groin Pain

**DOI:** 10.3390/medicina62091758

**Published:** 2026-09-12

**Authors:** Sergey Yu. Muraviev, Zakhar A. Akulov, Maria A. Sukhanova, Miroslava O. Pilipenko, Evgeniy A. Tarabrin, Zelimkhan G. M. Berikkhanov, Aleksey G. Kotelnikov, Andrey M. Nikolaev, Vadim S. Razumovsky, Milena Yu. Ivanova, Sara Nourmahal, Ruoran Xia, Vladislav S. Rakintsev, Alexey L. Shestakov

**Affiliations:** Department of Hospital Surgery No. 2, Sechenov First Moscow State Medical University (Sechenov University), Moscow 119048, Russia; muravev_s_yu@staff.sechenov.ru (S.Y.M.);

**Keywords:** osteitis pubis, periostitis, inguinal hernia, chronic postoperative inguinal pain, mesh fixation, pubic tubercle

## Abstract

*Background and Objectives*: Severe chronic postoperative inguinal pain (CPIP) affects 2–5% of patients after hernia repair, yet most published explanations remain neuropathic. The pubic periosteum, including the tubercle and adjacent symphyseal zone, lies within the operative field and may be engaged by medial mesh fixation. Its contribution to CPIP has not been tested. This narrative review brings together anatomical, imaging and clinical evidence and defines a hypothesis that can be examined prospectively. *Materials and Methods*: PubMed/MEDLINE, Embase and the Cochrane Library were searched without date restriction, with the final search run in April 2026. Thirty-three publications met the eligibility criteria. Evidence was classified using the 2011 OCEBM Levels of Evidence, with indirectness recorded when the population, exposure or outcome did not match the review question. The review was narrative, so no PRISMA flow diagram or formal risk-of-bias assessment was undertaken. *Results*: Direct evidence is limited. Among 1633 patients in one retrospective study, six underwent reoperation for severe late pain, and two had a staple placed in the periosteum. Pain resolved after staple removal. Two further cases of MRI-confirmed osteitis pubis followed laparoscopic repair with a titanium tacker. CT found pubic spurs in 17 of 20 patients, although their clinical meaning was uncertain. Fixation trials, CPIP reviews and groin-pain imaging studies repeatedly examined adjacent outcomes but did not define a periosteal endpoint. *Conclusions*: Pubic periosteal pathology is a plausible and testable pain generator, not an established cause of CPIP. Prospective studies should pair preoperative MRI and standardised pain mapping with explicit recording of fixation at the pubic tubercle. This design would show whether periosteal findings identify a distinct pain phenotype and whether penetrating fixation modifies its course.

## 1. Introduction

More than 20 million inguinal hernia repairs are performed worldwide each year [1]. Prosthetic mesh has reduced recurrence, but severe CPIP still affects 2–5% of operated patients [2]. The resulting loss of quality of life concerns hundreds of thousands of people.

The prevailing understanding of this pain is built on nerve injury. Systematic reviews of the surgical management of CPIP examine neurectomy, nerve blocks, pulsed radiofrequency therapy and neurostimulation [2,3]. Other sources of pain, osseous ones among them, fall outside their scope. One review notes that nociceptive pain may be related to pubic periostitis, but the observation is reflected neither in its methods nor in its conclusions [3].

Sports medicine has built a separate part of the literature on osteitis pubis. Its systematic reviews address treatment, rehabilitation and shockwave therapy almost entirely in athletes [4,5,6,7]. None included general surgical patients with a true inguinal or femoral hernia.

Hernia surgery and sports medicine describe the same anatomical neighbourhood from opposite sides. Surgeons work at the pubic tubercle but rarely interrogate the bone. Sports physicians characterise symphyseal pathology but seldom consider a true inguinal or femoral hernia. The periosteal lesion is consequently visible to each field only in fragments.

Accordingly, the clinically relevant question is whether the source of pain can be distinguished before surgery when pubic periosteal pathology coexists with an inguinal or femoral hernia. In a patient with a demonstrable hernia and medial pubic pain, symptoms attributed to the hernia may arise partly, or even predominantly, from the pubic symphysis and its periosteal-entheseal complex. If this overlap is missed, pain may persist after an anatomically successful repair. The objective of this review was therefore to examine that diagnostic overlap and determine how pre-existing pathology and fixation-related injury could be tested within a single clinical model.

## 2. Materials and Methods

We searched PubMed/MEDLINE, Embase (Ovid) and the Cochrane Library (CDSR and CENTRAL) without date restriction. The final search was run in April 2026. Search terms were grouped into hernia, pubic pathology and pain or outcome concepts. The reference lists of eligible papers, the 2018 HerniaSurge guidelines and publications from the Abdominal Core Health Quality Collaborative (ACHQC) registry were screened by hand.

We considered English-language original studies of any design, systematic reviews, meta-analyses and case reports. Two authors independently screened titles and abstracts and resolved disagreements through discussion. Thirty-three publications were retained because they addressed the pubic bone, mesh fixation or CPIP. The 2011 Oxford Centre for Evidence-Based Medicine (OCEBM) Levels of Evidence were applied according to study design and the clinical question addressed. Evidence was marked as indirect when the population, exposure or outcome did not match the review question. The review was narrative, so no PRISMA flow diagram or formal risk-of-bias assessment was undertaken.

**Figure 1:**
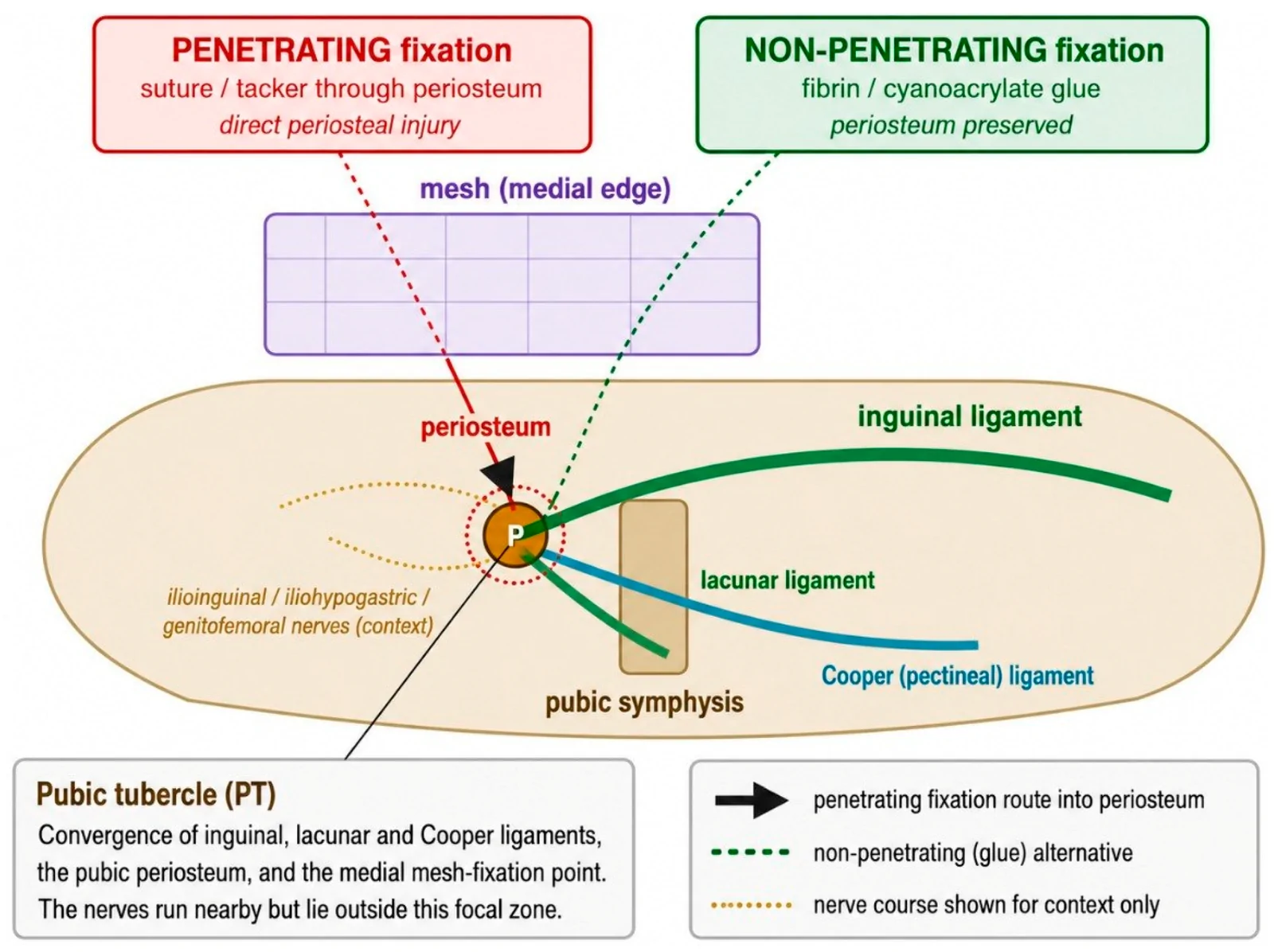
Anatomical convergence at the pubic tubercle. The inguinal, lacunar, Cooper’s and Henle’s ligaments meet the periosteal-entheseal complex beside the medial mesh margin. A suture or tacker can cross this zone, whereas glue remains superficial. Nearby groin nerves are shown for orientation. The figure separates direct mechanical contact from the proposed biological response.

**Figure 2:**
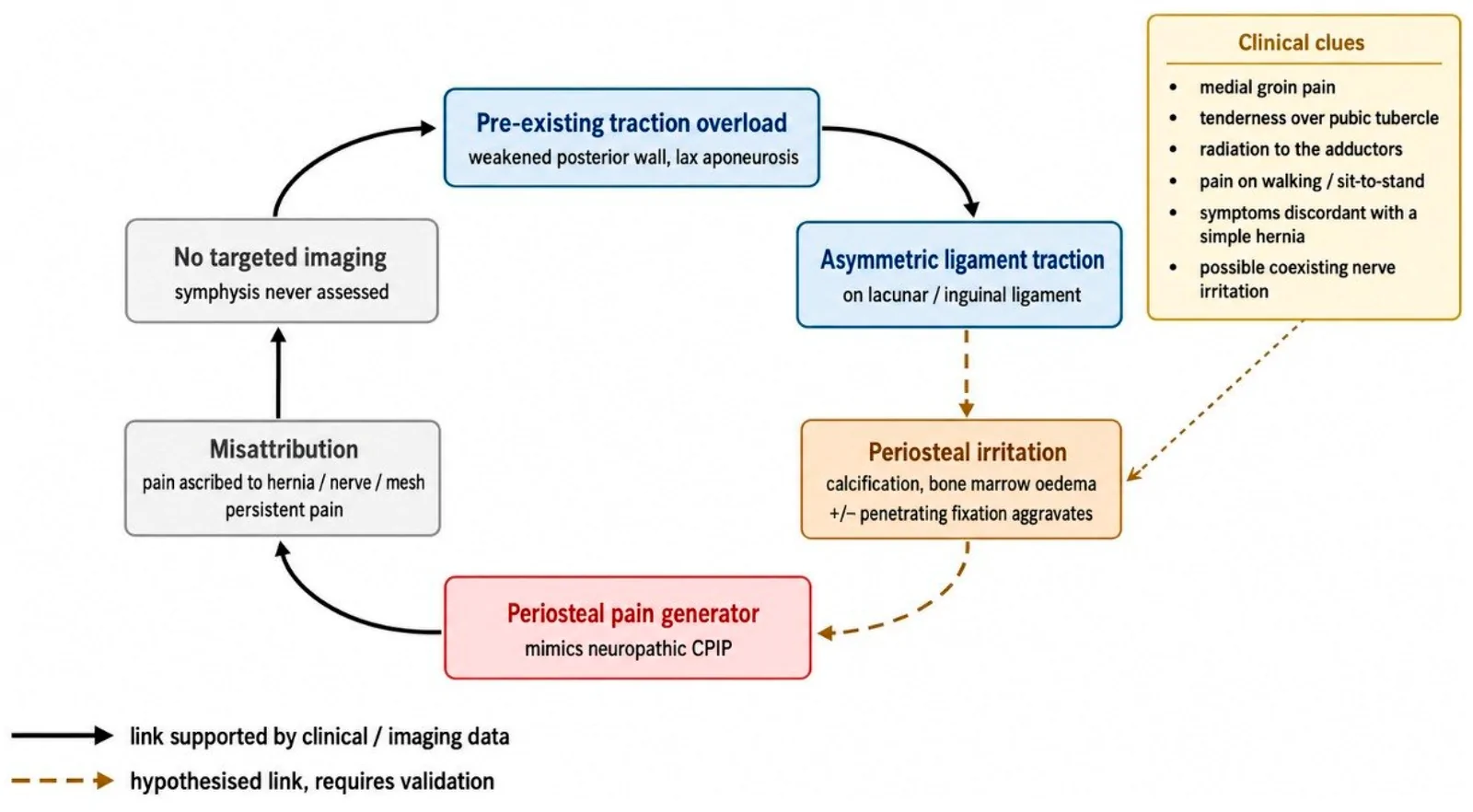
Proposed cycle of unrecognised periosteal pain in hernia and CPIP. Posterior-wall weakness may produce asymmetric ligament traction, periosteal or entheseal irritation, calcification and bone marrow oedema. Penetrating fixation could add a second insult. Pain may then be attributed to the hernia, a nerve or the mesh, while the absence of targeted imaging prevents correction of the initial diagnosis. Solid arrows indicate relationships supported by clinical or imaging observations. Dashed arrows indicate hypotheses that require testing.

For quantitative surgical comparisons, we extracted pooled estimates reported by existing reviews and did not perform a new meta-analysis because their trial sets overlap. Figure 1 and Figure 2 were redrawn in Python 3.12 with Matplotlib 3.10.8 and exported at 300 dpi.

## 3. Anatomical and Pathomechanical Basis

The pubic tubercle is the medial attachment point of the inguinal ligament (Figure 1). The fibres of the external oblique aponeurosis that form this ligament are in immediate contact with the periosteum of the symphysis. Repetitive loading of the symphyseal disc, characteristic of running and of kicking and rotational sports, accelerates its degeneration, leading to pelvic instability and microfissuring along the osteochondral junction with avulsion of the periosteum from the pubic ligaments [8]. The standard approach in anterior inguinal hernia repair traverses this same anatomical region.

The tubercle and symphysis are adjacent but not interchangeable. The pubic tubercle lies roughly 2–3 cm lateral to the symphysis and receives the medial mesh fixation, whereas osteitis pubis is described as a parasymphyseal process [4]. The periosteum of the superior pubic ramus is continuous between these sites. The inguinal, lacunar, Cooper’s and Henle’s ligaments, together with rectus abdominis and the adductors, form a connected fibroperiosteal complex. Injury or sustained traction at one insertion could therefore produce tenderness and reactive change nearby. This mechanism remains unproven because no study has tracked a periosteal reaction from the tubercle to the symphysis.

The term periostitis may itself be too narrow. The relevant lesion could lie in the periosteum of the pubic tubercle, the enthesis of Henle’s ligament (ligamentum interfoveolare), or both contiguous tissues. Those substrates occupy the same small operative zone but do not imply the same mechanism or treatment. Imaging and, where clinically justified, histopathology must separate them before a fixation-specific recommendation can be made.

Lichtenstein mesh fixation may begin with a suture in the periosteum of the pubic tubercle. The three-stitch technique states explicitly that the first stitch passes through the periosteum [9]. In laparoscopic repair, helical tacks can be placed close to the iliopubic tract and pubic bone. Fibrin glue applied at the tubercle was developed as a non-penetrating alternative [10].

The mechanistic distinction is simple. A suture, staple or tacker can pierce the periosteal zone, whereas glue cannot. What remains unknown is how often this contact produces inflammation, whether pre-existing enthesopathy amplifies the response and whether altered tissue can weaken fixation. No study has related implant pull-out, mesh migration or recurrence to preoperative periosteal findings.

A preoperative mechanism deserves separate consideration. The floor of the inguinal canal is formed medially by the superior pubic ramus and inguinal ligament, with the lacunar ligament between them [11]. Weakening of the posterior wall may redistribute tension across the inguinal and Henle’s ligaments. Repeated asymmetric traction at their insertions could promote thickening, inflammation and calcification. The pubic spurs found on CT in 17 of 20 patients [12] may represent this process, although the study did not test that explanation. Earlier CT herniography also identified pubic spurs in patients with groin pain and compared their origin with repetitive traumatic hypertrophy in Gilmore’s groin [13]. If the model is correct, part of the pain attributed to a hernia may precede surgery in the periosteal-entheseal zone later used for fixation.

Henle’s ligament is densely fused with the pubic region and sits between opposing forces. Rectus abdominis and the anterior abdominal wall pull cranially, while the hip adductors pull caudally. Repeated shear could carry posterior-wall microtrauma into chronic inflammation at the insertion and only later involve nearby nerve trunks. This sequence has not been demonstrated directly. It matters because routine assessment starts with nerves and may never test the tissue beneath the medial fixation point. Figure 2 maps the sequence and shows where the periosteal component disappears from routine diagnostic reasoning.

## 4. Direct Clinical Evidence

The only retrospective study that directly linked periosteal mesh fixation to severe chronic pain followed 1633 patients for nine years [14]. Six patients (0.37%) developed severe late pain 1.5–4 years after repair and underwent revision. None had early postoperative pain or wound infection. Four had ilioinguinal nerve entrapment. Two had a metallic staple placed directly in the periosteum of the pubic tubercle. Pain resolved completely after staple removal, with no recurrence during 6–44 months of follow-up. These selected cases cannot establish frequency, but they demonstrate a fixation-specific pain mechanism.

Two cases of osteitis pubis were reported after TEP repair with a titanium tacker [15]. MRI confirmed the diagnosis, and both patients improved with conservative treatment. The report establishes clinical plausibility but offers no denominator, comparator or preoperative imaging.

The SAGES manual includes a case of concurrent inguinal hernia and osteitis pubis and advises investigation and treatment of suspected osteitis before repair [16]. The recommendation rests on a case report, yet it is the only direct preoperative recommendation found in this review.

A case of pseudomonal osteomyelitis of the pubic symphysis one week after open hernia repair (erosion of the symphyseal margins on CT, *Pseudomonas* on blood culture, cure with ciprofloxacin) represents infectious rather than mechanical pathology but underlines the immediate proximity of the operative field to the bone [17].

A retrospective cohort of 100 patients with chronic groin pain and a mean follow-up of 13 years showed marked diagnostic heterogeneity [18]. The most frequent diagnoses were rectus abdominis atrophy (22), conjoint tendon tendinopathy (16), sports hernia (16), groin disruption (16), classical inguinal hernia (11), traumatic osteitis (5) and pubic avulsion fracture (4). Osteitis and classical hernia were therefore separate primary diagnoses in the same clinical population. Treating one without testing for the other risks leaving the original pain generator intact.

## 5. Imaging Findings Without an Addressee

Imaging deepens the disconnect. CT shows chronic osseous remodelling, including spurs and cortical calcification. MRI shows active features such as parasymphyseal bone marrow oedema and symphyseal degeneration. Without a shared protocol, the same region is described in different terms and at different stages of disease.

A prospective MDCT study found pubic spurs in 17 of 20 patients (85%) [12]. That frequency does not establish disease. The authors considered partial ossification at the pectineus insertion and called for a larger sample. Earlier CT herniography found bony spurs in two of 51 patients with clinically indeterminate hernias and groin pain and identified them as a possible pain source [13]. Neither study linked the finding to a standardised clinical phenotype.

A multidisciplinary imaging review proposed “pubic overload” in place of “osteitis pubis” and defined its MRI pattern as parasymphyseal bone marrow oedema with degeneration [11]. It also stressed that several causes of groin pain may coexist and that radiologists, hernia surgeons and orthopaedic surgeons should read the findings together.

Postoperative CT reviews focus on pseudomasses, seromas, haematomas and infection [19]. They do not list periosteal reaction as either an expected finding or a complication, although CT can depict chronic osseous change.

Observations exist on both sides, yet the connecting analysis is missing. A radiologist may explain osseous change locally as ossification or stress response. A surgeon may record pain at the same site but assign it to a nerve or the mesh. Neither account tests whether both findings belong to the same pain generator.

The evidence relevant to this hypothesis can be ordered by directness, from injuries observed after hernia repair to adjacent fields that touch on the question only in passing (Table 1).

## 6. Mesh Fixation and Periosteal Injury: Indirect Evidence

An umbrella review of 30 systematic reviews and meta-analyses found less persistent pain with glue than with sutures in open repair and with glue than with tacks in laparoscopic repair [20]. This difference is usually attributed to reduced nerve trauma. Yet non-penetrating fixation also avoids piercing the periosteal zone. None of the reviews recorded periosteal reaction, bone marrow oedema or the condition of the pubic tubercle.

A meta-analysis of 17 RCTs and 3150 hernias found no difference in chronic pain at one year between glue and sutures in Lichtenstein repair [21]. A second meta-analysis of 19 RCTs and 3578 patients found less pain one month after cyanoacrylate fixation but no difference in CPIP at one year [22]. The 2026 Cochrane review of laparoscopic repair included 35 RCTs and 4329 patients. It found no clear reduction in chronic pain risk or recurrence with non-penetrating fixation, and certainty was low or very low [1]. None of the three syntheses assessed bone.

These studies treat chronic pain as a single endpoint. They cannot show whether any difference arose from nerves, bone, entheses or another tissue. Separating those components is the necessary next experiment.

## 7. Sports Medicine and Hernia Surgery

The only systematic review of osteitis or osteomyelitis of the symphysis included 195 athletes and no patient with a concurrent inguinal hernia [4]. Its evidence was OCEBM level 4, and no randomised trial was found. Rehabilitation [5] and shockwave therapy [6] were also studied in athletic populations. A broader meta-analysis reported improvement in pain and function after shockwave therapy for bone marrow oedema but did not identify a hernia subgroup [7]. None of these studies tested coexistence with true inguinal or femoral hernia.

A review of 31 studies and 1571 patients with core muscle injury, athletic pubalgia or groin disruption mentioned osteitis as a concomitant condition but did not analyse it separately [25]. In this literature the pubic bone is described as the fulcrum between the abdominal muscles and hip adductors [26], and true inguinal hernia remains part of the differential diagnosis [27,28]. An editorial further noted that pubalgia describes pain at the pubis and that osteitis may require treatment alongside other components [29].

MRI may underestimate core muscle injury, while dynamic ultrasonography is increasingly used [30]. At the same time, asymptomatic MRI changes around the symphysis and adductors are common [31]. The “pubic inguinal pain syndrome” concept joins nerve compression, muscular imbalance and posterior-wall weakness but does not define a periosteal variable [32]. Reviews of return to sport likewise omit a separate periostitis analysis [33]. The ACHQC registry identified 60 patients with coexisting inguinal hernia and core muscle injury among 29,451 cases yet did not examine the symphysis [23]. A cohort of more than 12,000 patients with long-standing groin pain treated osteitis as one component of a multidisciplinary pathway, but its independent contribution could not be recovered from the published data [24].

## 8. The Knowledge Gap

No study has assessed the prevalence of pubic periosteal pathology in patients with inguinal or femoral hernia outside the athletic population. No meta-analysis of mesh fixation (more than 30 papers, thousands of patients) has included an osseous or periosteal outcome among the variables measured [20]. Periostitis has not been studied as a possible contributor to CPIP, and outcomes have not been compared between patients with and without preoperative osteitis of the symphysis. The contrast between what the existing literature measures and what it leaves unmeasured is presented in Table 2.

The literature misses the question in two ways. It studies athletes rather than general surgical patients, and it treats pain as a single outcome rather than separating neuropathic, osseous and entheseal components. No prospective cohort has recorded the preoperative state of the symphysis.

## 9. Rationale and Hypothesis

The evidence supports a coherent hypothesis but not a causal claim. Penetrating fixation contacts the periosteal zone, and selected clinical observations link a staple or tacker at that site to severe pain [14,15]. Glue is sometimes associated with less pain than penetrating fixation [20,21,22], but published explanations and outcome sets remain focused on nerves. Meanwhile, the frequency of asymptomatic symphyseal MRI findings [31] warns against treating imaging alone as disease.

Periosteal pain may remain invisible because its location and provoking factors overlap with other groin pain syndromes. It may be centred medially, worsen under load and radiate toward the adductors. After repair, the same symptoms can be assigned to nerve entrapment or the mesh. A spur or marrow oedema may be dismissed as incidental when imaging is not interpreted against a focused examination [12,13].

A broader model is therefore needed. Posterior-wall weakness may redistribute load to the pubic ligamentous insertions before surgery. In susceptible patients, that load could inflame the periosteal-entheseal complex. Repair removes the hernia but may leave the original pain generator in place, and penetrating fixation may add local trauma. This model explains persistent pain without presuming that every periosteal imaging abnormality is symptomatic.

The testable hypothesis is that a clinically defined subgroup of patients with inguinal or femoral hernia has pain arising from pubic periosteal or entheseal pathology before repair. Preoperative imaging-positive status and direct penetrating fixation should therefore be evaluated as separate predictors of postoperative pain.

## 10. Discussion

The practical question changes at each stage before, during and after repair.

Preoperatively, MRI of the pubic symphysis may be useful when pain is medial, radiates toward the adductors and is reproduced by walking, rising or focal palpation. Those features overlap with hernia and periosteal pain. Because asymptomatic MRI changes are common [31], a scan should be interpreted only alongside pain mapping, palpation of the tubercle and symphysis, and the relation of symptoms to a cough impulse. Where examination and imaging converge, a short period of load modification and targeted rehabilitation can test whether the medial pain phenotype changes before repair. This proposal is extrapolated from sports medicine and should not be treated as an established hernia protocol.

Intraoperatively, the biologically important variable may be the fixation method rather than access alone. Open repair permits direct exposure and palpation. Laparoscopic and robotic repair offer a posterior view and, in the robotic setting, greater instrument articulation. None of these advantages neutralises an inflamed fixation target. A non-penetrating strategy may be reasonable when periosteal pathology is confirmed, but the existing evidence supports only lower pain in some pooled comparisons, not a periosteal mechanism [10,20,21,22]. Access and fixation should therefore be analysed separately.

Postoperatively, persistent pain without recurrence, infection or a convincing neuropathic source should prompt consideration of the symphysis. If imaging and examination support periosteal involvement, load restriction, anti-inflammatory treatment and graded abdominal or adductor rehabilitation may be attempted before revision. Shockwave therapy has supportive evidence in adjacent populations [6,7], not in hernia-specific cohorts. Table 3 converts these proposals into a selective research pathway.

The operative comparison should follow the biology. Posterior-wall mechanics and the identity of the lesion come first, fixation second, and access third. Table 4 therefore compares access and fixation by their contact with the pubic zone rather than ranking operations.

Inflamed or remodelled tissue may also provide a less reliable fixation bed, creating a theoretical route to mesh displacement and recurrence. This possibility is clinically important but has not been measured. It is included as an endpoint in Table 5, not as a demonstrated consequence of periostitis.

Published comparisons can answer only part of the question. Their trial populations were not screened for periosteal disease, and several reviews reuse the same RCTs. We therefore present reported pooled estimates side by side and do not combine them again.

A formal preoperative-to-postoperative pain analysis is not supported by the available studies. Most reviews report postoperative group comparisons without a pooled baseline score, while direct periosteal reports describe selected cases. Table 6 shows what can be compared and where the data stop.

These data justify a staged study design. A prospective cohort should first define the imaging-positive pain phenotype with the same instrument before and after repair. Only then can a fixation trial test whether avoiding penetration of that zone changes pain or recurrence.

Four actions are ready for prospective testing:

• In patients with atypical medial pain, record a standardised pain map, examination findings and preoperative MRI features before attributing symptoms to the hernia.
• Record the location and mode of mesh fixation, together with periosteal reaction, as prespecified variables in fixation trials.
• Follow imaging-positive and imaging-negative patients prospectively with the same pain instrument before and after repair.
• After defining the phenotype, randomise imaging-positive patients to penetrating or non-penetrating fixation and measure pain, quality of life and recurrence.

## 11. Limitations

Direct evidence linking periostitis to inguinal hernia and CPIP does not exceed OCEBM level 4. No randomised trial has tested the proposed relationship. The retrospective revision series included six patients among 1633 repairs [14]. The TEP report described two cases [15], and the report of concurrent hernia and osteitis described one [16]. These observations cannot estimate risk in the wider hernia population.

Indirect evidence comes from methodologically stronger syntheses of fixation and pain outcomes [1,20,21,22]. Yet none prespecified a periosteal endpoint, so any mechanistic interpretation rests on anatomy and association. The present review is narrative and includes no formal risk-of-bias assessment. The search was limited to English-language publications.

The scale of the omission is itself informative. Thirty meta-analyses and thousands of participants were analysed without a bone-specific outcome.

## 12. Conclusions

Pubic periosteal pathology or the sesamoid band (still unknown) remains an unproven cause of CPIP and warrants a place in the differential diagnosis. The pubic tubercle lies at the intersection of ligamentous traction and medial mesh fixation. Direct reports show that a staple or tacker placed in the periosteal region can produce severe pain, while imaging studies and fixation trials repeatedly measure adjacent phenomena without recording a periosteal outcome.

The decisive next step is a prospective cohort with preoperative MRI, standardised pain mapping and stratification by fixation method. A randomised fixation study in imaging-positive patients should follow only after the phenotype is defined.

Until then, selective assessment of the symphysis in patients with atypical medial pain and explicit recording of periosteal findings are more defensible than assigning every persistent pain syndrome to a nerve or to the mesh.

## Figures and Tables

**Table 1:** Evidence map linking pubic periosteal pathology, inguinal hernia and chronic postoperative inguinal pain.

Evidence Domain	Representative Sources	Population	Key Finding	Directness	Principal Limitation
Periosteal injury after hernia repair	Delikoukos [14]; Marwah [15]; Al-Enazi [16]; Mader [17]	Operated hernia patients	A staple or tacker in the periosteum and osteomyelitis after repair caused chronic pain that resolved after removal of the fixation device	Direct	Case reports and small series. OCEBM level 4 evidence, with mechanism-based inference at level 5
Imaging of the pubic bone in groin pain	Cherian [12]; Markos [13]; Plumb [11]	Patients with groin pain and with hernia	Pubic spurs and parasymphyseal oedema are common and are proposed as a source of pain	Direct (imaging)	Small samples; uncertain clinical significance
Mesh fixation literature	Techapongsatorn [20]; Rancke-Madsen [1]; Phoa [21]; Trisca [22]	More than 10,000 patients in pooled RCTs	Non-penetrating fixation is associated with less chronic pain	Indirect	No periosteal or osseous outcome measured
Sports medicine/ osteitis pubis	Choi [4]; Cheatham [5]; Rhim [6]; Haußer [7]	Athletes only	Osteitis pubis is treatable but has been studied outside the hernia population	Adjacent	No subgroup of general surgical patients with hernia
Registries and concomitant groin pathology	Ellis (ACHQC) [23]; Garvey [18]; Santilli [24]	Large cohorts	Hernia and core muscle or groin pathology frequently coexist	Contextual	No subanalysis of the linking factors

**Table 2:** Measured and unmeasured outcomes in the literature on inguinal hernia, mesh fixation and CPIP.

Body of Literature	Outcomes or Response Domains Reported	Periosteal or Osseous Assessment	Consequence of the Omission
Mesh fixation reviews [1,20,21,22]	Recurrence, acute and chronic pain, operating time and complications	Absent from all studies	The osseous component is absorbed into overall chronic pain and cannot be separated from the neuropathic component
Reviews of CPIP management [2,3]	Change in pain after neurectomy, nerve block, radiofrequency treatment or neurostimulation	Not differentiated	Treatment is directed at the nerve by default
Reviews of osteitis pubis [4,5,6,7]	Pain, function, return to activity and response to rehabilitation or shockwave therapy	Studied, but with no hernia subgroup	Findings cannot be assumed to apply to general surgical patients
Registry data [23]	Frequency and management of coexisting inguinal hernia and core muscle injury	No separate analysis of the symphysis	Coexistence is recorded but not characterised
Guidelines and consensus documents	Recurrence, acute and chronic pain, complications and outcomes by operative technique	No targeted assessment of the symphysis	The surgeon is not instructed to assess the state of the bone
Femoral hernia literature	No dedicated outcome set	Absent	An anatomically adjacent region remains unstudied

For femoral hernia the volume of data is effectively nil, even though the femoral ring is anatomically adjacent to the pubic crest (Cooper’s ligament attaching to the superior pubic ramus).

**Table 3:** Selective research pathway for hernia patients with atypical medial pain.

Stage	Trigger	Action	Rationale	OCEBM Level and Applicability	What Requires Verification
Preoperative	Medial pain radiating to the adductors, focal tenderness or symptoms not explained by the hernia alone	Map pain and examine the tubercle and symphysis; consider MRI when findings converge	Hernia and periosteal pain overlap; MRI may show subchondral oedema	OCEBM levels 3–4, indirect [11,31]	Prospective study of diagnostic accuracy against clinical and operative findings
Intraoperative	Periosteal or entheseal abnormality on imaging or at surgery	Record fixation location and device; consider non-penetrating fixation	Direct penetration is avoidable, although a periosteal benefit is unproven	OCEBM level 1, indirect [20], and level 4 [10]	RCT stratified by preoperative imaging with a periosteal outcome
Postoperative	Persistent pain not explained by nerve injury, recurrence or infection	Examine the symphysis, consider MRI and use graded conservative care before revision	Periosteal pain may mimic CPIP, while sports-medicine treatments are indirect	OCEBM levels 1–4, indirect [2,3,6,7]	Prospective cohort with standardised pain mapping and imaging

This pathway is a research framework, not a clinical guideline. It is intended for selected patients and requires prospective validation.

**Table 4:** How operative access and fixation engage the pubic periosteal-entheseal zone.

Approach and Fixation	View and Handling of the Pubic Zone	Relation to Periosteum	Potential Advantage	Main Uncertainty or Risk
Open anterior repair with sutures [9,14]	Direct exposure and palpation of the tubercle	The medial suture can enter the periosteal zone	Controlled placement under direct vision	Penetrating injury and nerve entrapment are possible. Preoperative periosteal status was not recorded
Open anterior repair with glue [10,20,21,22]	The same anterior exposure with adhesive fixation	No puncture of the fixation surface	Avoids direct penetration and may reduce early pain	Long-term pain estimates are inconsistent and no periosteal outcome was measured
TAPP or TEP with tacks or staples [1,15,20]	Posterior view of Cooper’s ligament and the tubercle without external palpation	A device may penetrate tissue adjacent to the pubic bone	Broad posterior view and familiar mesh overlap	Case reports describe osteitis. The causal risk cannot be quantified
TAPP or TEP with glue, self-fixating or preformed 3D mesh, or no fixation [1,20]	Posterior dissection without a penetrating device at the tubercle	Avoids direct perforation of the periosteal zone	Separates mesh placement from penetrating fixation	Pain and recurrence results are not stratified by preoperative periosteal disease
Robotic posterior repair	Posterior view with articulated instruments and precise placement	May use penetrating or non-penetrating fixation	Precise dissection and device placement	No direct periosteal comparison exists. Technical precision does not neutralise an inflamed target

**Table 5:** Reported pain and recurrence estimates for penetrating and non-penetrating fixation.

Evidence Set	Comparison	Pain Estimate	Recurrence Estimate	Periosteal Interpretation
Cochrane review, 35 RCTs and 4329 patients [1]	Non-penetrating versus penetrating fixation in laparoscopic repair	Chronic pain risk RR 0.62 (95% CI 0.35–1.07, 7 RCTs, *n* = 553); chronic pain score MD −0.34 (95% CI −0.56 to −0.11, 7 RCTs, *n* = 639)	RR 0.75 (95% CI 0.33–1.67, 15 RCTs, *n* = 2165)	No clear difference in chronic pain risk or recurrence; certainty was very low, and bone was not assessed
Umbrella review, 30 systematic reviews and meta-analyses [20]	Glue versus sutures in open repair and glue versus tacks in laparoscopic repair	Glue favoured for persistent pain in both open and laparoscopic syntheses	No significant difference in open repair (*p* = 0.816) or laparoscopic repair (*p* = 0.946)	High overlap between reviews and no periosteal endpoint
Meta-analysis, 17 RCTs and 3150 hernias [21]	Glue versus sutures in open Lichtenstein repair	Chronic pain at 1 year OR 1.10 (95% CI 0.73–1.65, *p* = 0.65)	Early recurrence OR 1.11 (95% CI 0.45–2.76, *p* = 0.81); late recurrence OR 1.23 (95% CI 0.59–2.59, *p* = 0.59)	No pain or recurrence advantage was demonstrated, and periosteal status was unknown
Meta-analysis, 19 RCTs and 3578 patients [22]	Cyanoacrylate versus sutures in open Lichtenstein repair	Significant pain at 1 month was 2.37% versus 13.3%, OR 0.158 (95% CI 0.064–0.386, *p* < 0.001); one-year CPIP OR 0.96 (95% CI 0.72–1.30, *p* = 0.835)	One-year recurrence OR 0.68 (95% CI 0.25–1.80, *p* = 0.43)	Early pain favoured glue, but no durable CPIP difference or bone-specific assessment was reported

RR denotes risk ratio, OR odds ratio, MD mean difference, CI confidence interval and RCT randomised controlled trial. Estimates are reproduced from the cited syntheses and are not statistically independent because the underlying trials overlap. None included a periosteal or osseous outcome.

**Table 6:** Pain before and after repair as permitted by the published data.

Evidence and Intervention	Preoperative Pain State	Early Postoperative Pain	Late Pain or Response	Inference Limit
Open Lichtenstein repair with cyanoacrylate or sutures [22]	Groups did not differ in preoperative pain; a pooled baseline score was not reported	At 1 month, significant pain was 2.37% with cyanoacrylate and 13.3% with sutures, OR 0.158 (95% CI 0.064–0.386)	At 1 year, CPIP OR 0.96 (95% CI 0.72–1.30)	No paired change score and no preoperative imaging stratum
Laparoscopic repair with non-penetrating or penetrating fixation [1]	A pooled baseline pain score was not reported	Acute pain score MD −0.90 (95% CI −1.31 to −0.49, 16 RCTs, *n* = 1611)	Chronic pain risk RR 0.62 (95% CI 0.35–1.07); chronic pain score MD −0.34 (95% CI −0.56 to −0.11)	Very low certainty for chronic pain and no periosteal imaging
Anterior repair followed by staple removal [14]	Preoperative pain phenotype and bone imaging were not reported	The six revised patients had no early postoperative pain	Delayed severe pain occurred in two patients with a periosteal staple and resolved after removal in both	Selected reoperations, no comparator and no denominator for periosteal placement
TEP repair with a titanium tacker [15]	Preoperative osteitis was not documented	Postoperative osteitis was diagnosed in two cases	Symptoms resolved with conservative treatment in both	Case reports with no denominator, comparator or baseline imaging

## Data Availability

All data analysed in this review are drawn from the publications listed in the reference list. No primary data were generated.
